# A prospective comparative study to assess the risk of postoperative bleeding after dental surgery while on medication with direct oral anticoagulants, antiplatelet agents, or vitamin K antagonists

**DOI:** 10.1186/s12903-021-01868-7

**Published:** 2021-10-07

**Authors:** Mayte Buchbender, Nicola Schlee, Marco R. Kesting, Jannik Grimm, Jakob Fehlhofer, Andrea Rau

**Affiliations:** 1 Department of Oral and Maxillofacial Surgery, Friedrich-Alexander-University Erlangen-Nuremberg, Glückstrasse 11, 91054 Erlangen, Germany; 2 Department of Oral and Maxillofacial Surgery, Universitätsmedizin Greifswald, Greifswald, Germany

**Keywords:** Anticoagulant agents, Direct-acting oral anticoagulants, Hematological agents, Oral hemorrhages, Oral surgical procedures, Postoperative hemorrhages

## Abstract

**Background:**

The aim of this prospective study was to investigate the occurrence and severity of postoperative bleeding following dentoalveolar surgery in patients with uninterrupted anticoagulation therapy (AT).

**Methods:**

Patients receiving AT (vitamin k antagonist (VK), direct oral anticoagulants (DOAC) or antiplatelet therapy (APT) and in need of surgical intervention classified as A, B or C (single or serial tooth extraction, osteotomy, or implant placement) were studied between 2019 and 2021. A healthy, non-anticoagulated cohort (CG) served as a control group. The main outcomes measured were the frequency of postoperative bleeding, the classification of the severity of postoperative bleeding (1a, 1b, 1c, 2, 3), and the correlation with the AT surgical intervention classification.

**Results:**

In total, 195 patients were included in the study, with 95 patients in the AT group and 100 in the CG. Postoperative bleeding was significant in the AT group vs. the CG (*p* = 0.000), with a significant correlation with surgical intervention class C (*p* = 0.013) and the severity class of bleeding 1a (*p* = 0.044). There was no significant correlation with procedures of type A, B or C for the other postoperative bleeding gradations (1b, 1c, 2 and 3). There was a statistically significant difference in the occurrence of postoperative bleeding events between the DOAC/APT group and the VK group (*p* = 0.036), but there were no significant differences regarding the other AT agents.

**Conclusion:**

The continuation of anticoagulation therapy for surgical interventions also seems reasonable for high-risk interventions. Although significantly more postoperative bleeding occurs, the severity of bleeding is low. The perioperative management of anticoagulated patients requires well-coordinated interdisciplinary teamwork and detailed instruction of patients.

*Clinical trial registration* The study is registered (29.03.2021) at the German clinical trial registry (DRKS00024889).

## Introduction

### Background

Due to the increased prevalence of cardiovascular diseases, the number of long-term anticoagulated patients continues to increase worldwide [1]. The therapeutic management of such underlying diseases (e.g., apoplexy, atrial fibrillation, coronary heart disease, peripheral arterial occlusive disease, heart valve replacement or thrombosis) is an internal medicine issue [2, 3]. However, with increasing incidences of these diseases, anticoagulation therapy remains a main issue in oral surgery and dental surgical procedures [4]. In addition to the requirement of close interdisciplinary coordination between cardiologists, general practitioners and oral surgeons [5] to minimise the risk of potentially lethal thromboembolic events for the patients [6], dentist have to assess the risk of bleeding events in advance and individually for each surgical procedure and patient [7]. A general discontinuation of anticoagulation is no longer suggested according to current literature findings. In a review, it was reported that 22 embolic events occurred after the discontinuation or reduction of anticoagulation, and 6 of them had fatal endings; whereas, no fatal consequences were observed after postoperative bleeding had occurred with existing anticoagulation [8]. Even if the continuation of anticoagulation fortunately does not result in a life-threatening condition of the patient, discomfort from postoperative bleeding may still emerge from the surgical re-entry to stop bleeding, and even hospitalisation can occur [4]. The anticoagulation medication used in all classes intervenes differently in the coagulation cascade. Moreover, we know that the surgical intervention, respectively the intraoperative procedure possibly shows an effect on the postoperative bleeding events or postoperative sequalae [9].

Several authors investigated different AT medications and the occurrence of bleeding. In one study, 32 patients with bridging, 28 patients continuing to use vitamin K antagonists and 183 patients with simple antiplatelet therapy (APT) in the form of acetylsalicylic acid or clopidogrel were examined. A gelatine sponge was inserted, and tranexamic acid-soaked bite swabs were used as the primary haemostatic method. The frequency of postoperative bleeding did not vary significantly between the bridging group (8.1%) and the vitamin K group (9.1%). The frequency of postoperative bleeding in the APT group with acetylsalicylic acid (1.1%) and APT group with clopidogrel (4.2%) were comparable with that of healthy control patients (0.7%). No correlation with the type of surgical intervention was investigated, but it was concluded that anticoagulation can be continued under local haemostatic measures [10]. In a review from 2020 regarding the management of direct oral anticoagulants (DOACs), 9 studies were included that reported the frequency and severity of postoperative bleeding. The authors concluded that low-risk surgical interventions could be performed without interrupting the AT. If high-risk surgical intervention is to be performed, the authors recommended that the intervention should be performed, at the earliest, 24 hours after the last intake of medication. The authors have classified a low-risk situation as one to three tooth extractions or one to two implant placements, and a high-risk situation as 4 or more tooth extractions or more than two implant placements, and the studies were grouped according to that [11]. Nevertheless, when regarding the current state of evidence-based literature, and when including all anticoagulation medications regardless of their effect, one fact becomes apparent: postoperative bleeding does occur; however, it seems to be mostly minor so it is always concluded that the medication should be continued in so called low-risk procedures under local haemostatic measures. Furthermore, the classifications of intervention risks by the authors vary.

### Aim

However, it is also evident that not all studies have necessarily included the type of intervention, so this study analysed all types of AT patient cohorts in relation to the frequency of the postoperative bleeding events and the type of intervention (pre-classified as low, medium, or high-risk interventions) with the aim of deriving a possible risk assessment. We hypothesised that postoperative bleeding occurs mostly in high-risk interventions.

## Material and methods

The present study reports a monocentric prospective comparative clinical trial at the Department of Oral and Maxillofacial Surgery, University of Erlangen, Nuremberg. The patients included in this study underwent one of the anticoagulation therapies (AT) (direct oral anticoagulants, antiplatelet agents or vitamin K antagonists) at the Department of Oral and Maxillofacial Surgery, University of Erlangen-Nuremberg, from April 2019 to February 2021 (an inclusion period of 2 years). Ethical approval (No. 216_18B) was obtained from the Ethics Committee of the Medical Faculty at Friedrich-Alexander University, Erlangen-Nuremberg, and the guidelines of the Declaration Helsinki were followed. The study was also registered in the German clinical trial registry (DRKS00024889). All patients who had an oral surgical intervention (tooth extraction, osteotomies or implant placements) were included at the screening visit prior to surgery if they were older than 18 years, willing to participate, and had signed the surgical and ethical consent form. Patients with haemorrhagic diatheses, such as haemophilia, factor deficiency, von Willebrand-Jürgens syndrome, liver cirrhosis and thrombocytopenia, were excluded. Patients with incomplete documentation were not considered for the study. Moreover, patients with an international normalised ratio [12] > 3,5 were excluded. A control group (CG) with no anticoagulation therapy or nondrug-related coagulation was studied by retrospectively analysing patient data by screening the digital clinic documentation system (MCC®, Meierhofer AG, Munich, Germany) and the digital patient file (Soarian Clinicals®, Cerner Health Services, Erlangen, Germany) using the following key words: tooth extraction, tooth osteotomy, surgical intervention, tooth, bleeding event, anticoagulation and thromboembolic event. Patients in the study group were divided into the following treatment groups depending on the type and invasiveness of the planned procedure:

A. Single tooth extraction (simple extraction without raising a mucoperiosteal flap or damaging the marginal gingiva)
B. Serial tooth extraction (simple extractions of ≥3 teeth per quadrant without raising a mucoperiosteal flap or damaging the margin gingiva)
C. Serial tooth extraction (simple extractions >3 teeth per quadrant, osteotomies with raising a mucoperiosteal flap without periosteal incision and implant placements >1 per jaw)

Depending on the AT, the included patients were assigned to the following subgroups:

1. Vitamin k antagonist (VK)
2. Direct oral anticoagulants (DOAC)
3. Antiplatelet therapy (APT)

The study protocol consisted of minimally invasive treatment and the following primary local haemostatic measures were applied in each patient in the AT group:

1. Wound closure (adaptive suture of the extraction site) with resorbable Vicryl® 4-0 and 5-0 (Johnson &Johnson Medical GmbH, Norderstedt, Germany)
2. Tranexamic acid (Cyklokapron®, Pfizer Pharma GmbH, Berlin, Germany) with bite swab und local wound compression (instructions for the patients in the home environment)

In cases of secondary postoperative bleeding events, further measures were taken (bipolar electrocoagulation or inserting haemostatic filler materials into the extraction site (oxycellulose, collagen or porcine gelatine). After clarification with the patients’ general practitioners and cardiologists, the patients were asked to continue with their AT throughout the surgical intervention without stopping for surgery or even for a duration of several hours (especially in the DOAC group). The INR was monitored in the AT group. Patients in the control group were sutured only when mucoperiosteal flaps were raised or in cases of serial extractions.

Every surgical procedure was performed under local anaesthesia (Ultracain® DS; adrenaline 1:200,000; Sanofi-Aventis GmbH, Frankfurt, Germany). Endocarditis prophylaxis, based on the guidelines of the American Heart Association, was administered 1h prior to surgery if needed (a single shot of amoxicillin 2g or clindamycin 600mg). The surgical interventions were performed by one experienced oral surgeon (MB) or two oral surgical beginners (JG, JF) who were instructed prior to the study to apply the same measures outlined in the study protocol described above. Tooth extractions were performed without damaging the margin gingiva, intra-alveolar osteotomies were performed and in cases of flap elevation, mucoperiosteal flaps were performed with only one vertical incision and without periosteal incision.

Patients were instructed to protocol their postoperative bleeding events, and the extent of bleeding was categorized as follows:

- Slight postoperative bleeding on the day of surgery that independently sustained or through bite swabs at home
- Slight postoperative bleeding for up to three days after surgery that independently sustained or through bite swabs at home
- Slight postoperative bleeding more than three days after surgery that independently sustained or through bite swabs at home
- Moderate postoperative bleeding that required postoperative haemostatic measures by the surgeon
- Severe postoperative bleeding that required postoperative haemostatic measures by the surgeon and hospitalisation

The follow-up examination (wound inspection) was performed 10 days after surgery in all patients (AT and CG). Sutures were only removed if they disturbed the patients.

For both groups (AT and CG), the following parameters were recorded:

- Frequency and classification of the severity of postoperative bleeding events according to 1a, 1b, 1c, 2 or 3
- Surgical intervention and postoperative bleeding events according to A, B, C
- Correlation of postoperative bleeding events/surgical intervention and AT

For ensuring the comparability of the parameters, they were objectified by a previously defined scale of postoperative bleeding severity (1a, 1b, 1c, 2, 3) and the extent of surgical intervention (A, B, C) as described above. To keep the bias low, the surgical procedure was uniformly determined for all patients, and the operating surgeons were aligned before the procedure.

The bias that subjectively emanates from the included patients can certainly not be completely ruled out, but by instructing the patients to use the scale when assessing postoperative bleeding, an attempt was made to minimise this. The study size was statistically determined in advance. According to the literature, the expected postoperative bleeding rate was 3% in the control group and 17% in the study group. With a calculated power of 0.80 and a significance level of *p* < 0.05, a t-test calculation of 81 patients per group was necessary.

The primary outcomes measured in the study were the frequency and classification of postoperative bleeding in each group. Secondary outcomes were the type of surgery (single tooth extraction (simple extraction without raising a mucoperiosteal flap or damage of the marginal gingiva); serial tooth extraction (simple extractions of ≥ 3 teeth per quadrant without raising a mucoperiosteal flap or damage of the margin gingiva); and serial tooth extraction (simple extractions > 3 teeth per quadrant, osteotomies with raising a mucoperiosteal flap without periosteal incision and implant placements > 1 per jaw) and the correlation of postoperative bleeding events in relation to the AT.

### Statistical analysis

Statistical analysis of the collected data was performed using SPSS, Version 25 software (IBM, Armonk, New York, USA). The non-parametric chi-square test, in accordance with Pearson, was carried out to analyse a possible dependency between two study parameters.

Due to some small numbers in the groups, Fisher’s exact test was applied in addition. To check the equality of the variances of the investigated groups, the Levene test was carried out. The mean values of the test parameters were compared using the *t* test.

The level of significance was set to *p* < 0.05 in all tests.

## Results

### General patient data

As a result of the data analysis, a total of 195 patients (AT n = 95 with 62 men and 33 women, CG n = 100 with 58 men and 42 women) were included in the study and distributed into the subgroups of the AT group as follows: VK n = 17 (m = 9; w = 8); DOAC n = 27 (m = 18; w = 9); APT n = 51 (m = 35; w = 16) with no significant difference (*p* = 0.308). In the DOAC group, the active agents were divided as follows: apixaban n = 10, edoxaban n = 5, rivaroxaban n = 11 and dabigatran n = 1. In the APT group, 43 patients were treated with ASS®, 7 with clopidogrel and 1 with ticagrelor. In the VK group, the mean INR was 2.2 (maximum 3.0, minimum 1.5). The indications and basic diseases in the AT group are listed in Table 1. The overall mean age in the AT group was 73.29 and 62.01 in the CG (*p* = 0.000). The overall mean age in the subgroups of the AT group was 69.65 in the VK group, 77.96 in the DOAC group, 72.90 in the APT group with no significance.

**Table 1:** Showing the number of patients in correlation to the diseases and indications of the anticoagulation therapy AT

Indication disease for AT	Number of patients (n)
Hypertension	39
Coronary heart disease	8
Stent implant	20
Chronic heart failure	29
Artificial heart valve	5
Apoplexy	11
Endocarditis	2
Bypass	10
Cardiac pacemaker	3
Thromboses	2
Peripheral arterial disease	1
Aortic aneurysm	1
Heart valve vitias	4
Carotic stenosis	1
Myocardial infarction	13
Myocarditis	1
Defibrillator	1
Arrythmia	1
Koagulopathie	1
Cerebral microangiopathy	1
n.a.	10
Embolism	4

### Frequency and classification of the severity of postoperative bleeding events

Postoperative bleeding occurred in 25 out of 195 patients, with 25 in the AT group and 2 in the CG, with a significance of *p* = 0.000 for the AT group vs. CG. The distribution according to the severity of postoperative bleeding is shown in Table 2. In 92.6% of the patients, postoperative bleeding was assigned to 1a and 66.6% to 1b+1c in the AT group. Postoperative bleeding of severity grades 2 and 3 occurred in only one patient each. The patient with a severity grade 2 report was a 72-year-old man who was medicated with rivaroxaban because of hypertension and ten different co-medications. After 4 teeth were extracted, the patient reported postoperative bleeding (the patient constantly noticed blood in the saliva) for one week after the surgical intervention. After one week, the coagulum was removed and a new suture were performed. Afterwards, there was no further postoperative bleeding. The patient with the severity grade 3 report was a 72-year-old man who was medicated with dual anticoagulation therapy in the form of apixaban and clopidogrel because of stent implants and myocardial infarction, and he had 8 different co-medications. The surgical intervention consisted of extracting 3 teeth, and on the day of the surgery, the patient experienced postoperative bleeding. He had to be hospitalised, and an acrylic plate was used and the interruption of the apixaban medication were conducted. After one day, no further bleeding was recorded, and the patient could be discharged from the hospital. No significant correlation between postoperative bleeding and age was found (*p* = 0.370), and no correlation between postoperative bleeding and sex was found (*p* = 0.349). Moreover, no correlation between co-medication or basic disease and postoperative bleeding was found.

**Table 2:** Showing the postoperative bleeding events within the different groups (AT group and CG) in relation to the severity of postoperative bleeding grade 1a, 1b, 1c, 2 and 3

	AT Group (n)	CG (n)
1a	16	1
1b	5	1
1c	2	–
2	1	–
3	1	–
Total	25	2

### Surgical intervention and postoperative bleeding events

**Fig. 1:**
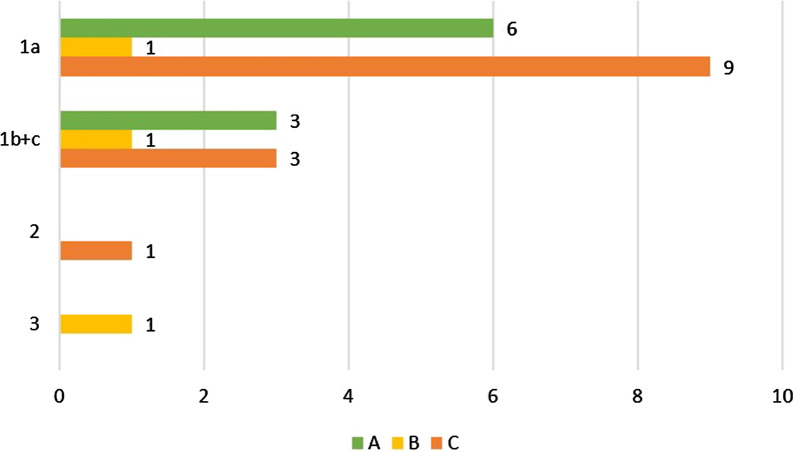
Showing the correlation of the surgical intervention (A, B, C) to the severity of postoperative bleeding (1a, b, c, 2, 3).

In a total of 195 surgical interventions, group A surgical procedures were performed in 92 of the cases, group B in 42 cases and group C in 61 cases. The distribution of surgical intervention according to the AT and CG groups is shown in Table 3, with no significant relationship between the groups and surgical interventions (*p* = 0.401). Regarding postoperative bleeding in correlation with the surgical intervention, there was a statistically significant difference between postoperative bleeding and interventions in group C (*p* = 0.013). The correlation of the surgical intervention (A, B, C) to the severity of postoperative bleeding (1a, b, c, 2, 3) is shown in Fig. 1, with a significance of postoperative bleeding of severity 1a in the AT group correlating to surgical intervention C (*p* = 0.044). There was no significant correlation with procedure type A, B or C for the other postoperative bleeding gradations 1b, 1c, 2 and 3. In total, there were 15 postoperative bleeding events in group C of the n = 31 surgical interventions, and n = 25 postoperative bleeding events in the AT group, with detailed surgical intervention of procedure type C and is shown in Table 4. In the CG, n = 2 postoperative bleeding events were assigned to group A.

**Table 3:** Showing the numbers of interventions according to the procedure type A, B and C within the different groups (AT group and CG)

	AT Group (n)	CG (n)
A (Single tooth extraction (simple extraction without raising a mucoperiosteal flap or damage of the marginl gingiva)	47	45
B (Serial tooth extraction (simple extractions of ≥3 teeth per quadrant without raising a mucoperiosteal flap or damage of the margin gingiva)	17	25
C (Serial tooth extraction (simple extractions >3 teeth per quadrant, osteotomies with raising a mucoperiosteal flap without periosteal incision and implant placements >1 per jaw)	31	30

**Table 4:** Showing the detailed surgical intervention of procedure type C with n=15 postoperative bleeding events within

n	Surgical intervention
3	Extraction of 4 teeth
2	Extraction of 5 teeth
1	Extraction of 6 teeth
1	Osteotomy of 1 tooth
2	Osteotomy of 3 teeth
2	Extraction of 1 tooth and Osteotomy of 1 tooth
1	Extraction of 6 teeth and Osteotomy of 1 tooth
1	Extraction of 2 teeth and implant uncovering of 2 implants
1	Uncovering of 3 teeth and Osteotomy of 1 tooth
1	Implant placement of 4 implants
Total: 15	

### Correlation of postoperative bleeding events/surgical intervention and AT

**Fig. 2:**
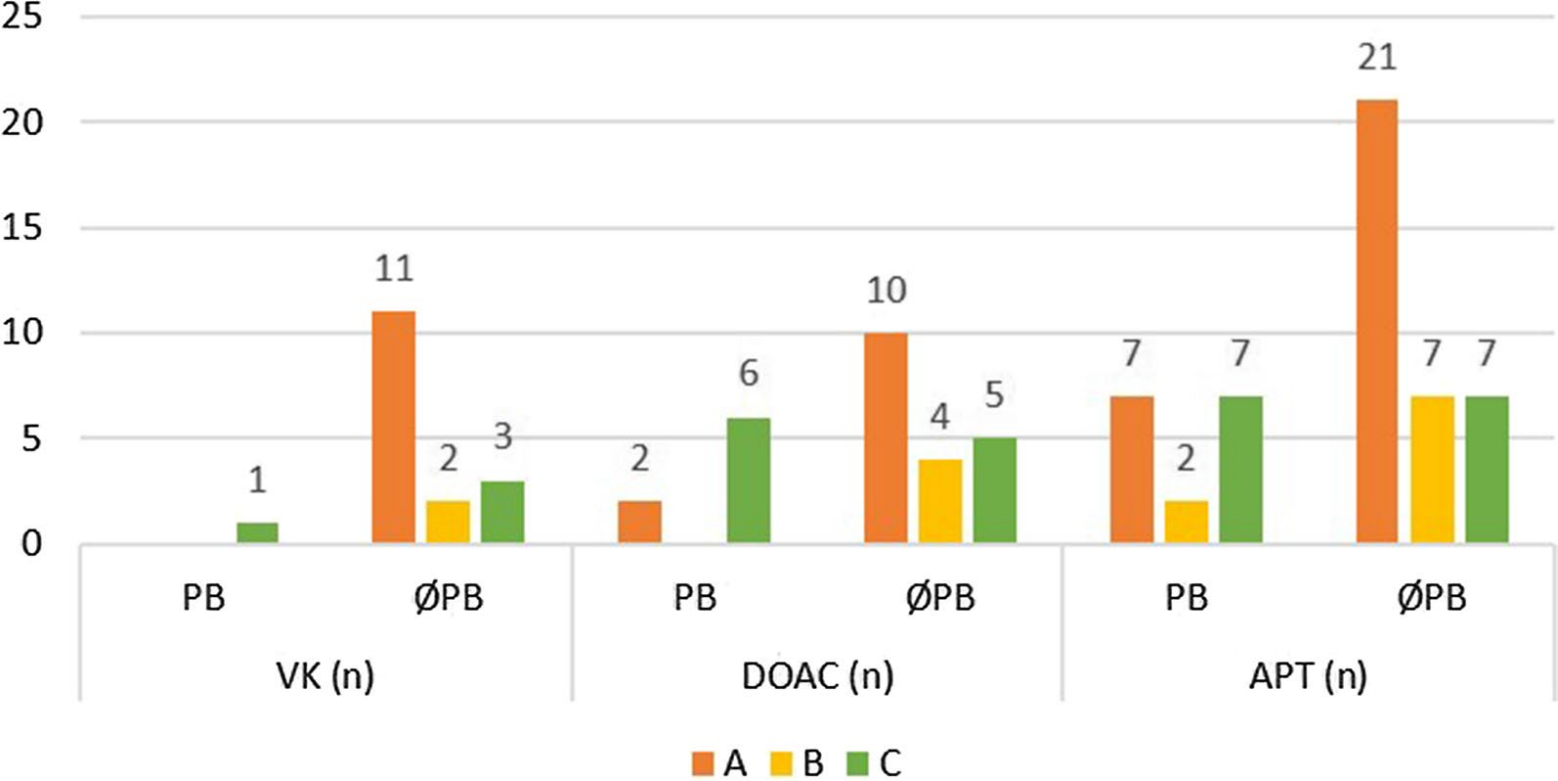
Showing the correlation and number of patients of intervention type A, B and C and the subgroups of AT (VK, DOAC, APT) with either postoperative bleeding (PB) or no postoperative bleeding (ØPB)

The correlation of the intervention type and AT is shown in Fig. 2. In the VK group, only one patient had postoperative bleeding that correlated with surgical intervention type C, which occurred in a total of 17 interventions. In the APT group, 16 (31.4%) postoperative bleeding events occurred (7 in correlation with surgical intervention types A and C and 2 in correlation with intervention type B) in a total of 51 interventions. In the DOAC group, 8 (29.6%) postoperative bleeding events occurred (2 in correlation with the surgical interventions type A and 6 in correlation with type C) in a total of 27 surgical interventions. There was a statistically significant difference in the occurrence of postoperative bleeding events between the DOAC/APT group and the VK group (*p* = 0.036). No significant difference was shown between VK/DOAC group and the APT group (*p* = 0.371). Moreover, no significant difference was shown between the APT/VK group and the DOAC group (*p* = 0.801).

## Discussion

Anticoagulated patients continue to pose a challenge, especially for surgical interventions [13]. Discontinuation of AT can indeed lead to thromboembolic events with potentially lethal outcomes [6, 14], so the main question always remains the same when it comes to dental surgery: when does the patients need to pause or what are the consequences if it is not paused? To answer this question, the type of surgery and the associated risk of bleeding must be assessed in advance, and the surgical approach must be planned in detail. There are several guidelines which offers surgeons different therapy approaches regarding the AT [7], although bleeding can certainly never be totally avoided. However, this lead practitioners and patients to the question: how drastic is this bleeding? To evaluate the risk of postoperative bleeding, the corresponding procedures within this study were categorized into low (A), medium (B) and high risk (C), with no patients pausing the AT. Postoperative bleeding was significantly more frequent in the AT group compared to the healthy group, with 25 postoperative bleeding events in 95 AT patients and these were significantly correlated to the C procedure type. Furthermore, the intensity was pre-classified (1a, 1b, 1c, 2 and 3), with postoperative bleeding events within the AT group and C type procedures significantly belonging to the intensity group 1a. 92.6% of the 25 postoperative bleeding events were significantly assigned to the intensity grade 1a, which we classified as slight postoperative bleeding on the day of surgery that was sustained individually or through bite swabs.

However, postoperative bleeding can be burdensome for the patient and may complicate wound healing. Primary wound management, in terms of adaptive sutures, as well as handling with bite swabs that are additionally soaked with tranexamic acid as local haemostatic measure, does not ultimately reduce the number of absolute bleeding events that occur in AT patients, but it certainly reduces the intensity and duration. Thus, studies that have a surgical approach similar to ours (minimally invasive surgical approach, adaptive wound suturing, and bite block swabs) also show that significantly more bleeding occurs in AT patients than in healthy individuals [16]. Primary wound care by suture was also investigated in a study with APT and VK in 108 patients, and postoperative bleeding occurred in only one case [17]. If primary haemostasis is not achieved by using bite swabs, other authors also recommend suture placement [10].

Within this study we investigated 17 surgical interventions with one postoperative bleeding event in the VK group and 51 interventions with 16 postoperative bleeding events in the APT group. Considering vitamin K antagonists, bridging has been found as not indicated according to the current state of the art practices, and with local haemostatic measures and an adjusted INR of 2–3.5, dentists can derive treatment recommendations to perform dental surgery in a controlled manner [4, 18, 19]. This is based on the fact that in previous studies, the incidence of postoperative bleeding events significantly increased in patients bridged with heparin. In this study, bridging was not investigated, but patients who were medicated with vitamin K were included with a mean INR of 2.2. It was also shown that patients had significantly more frequent postoperative bleeding events under DOAC therapy or APT; however, the VK group was the smallest here, with n = 17, so this has to be assessed critically. Moreover, the frequency of postoperative bleeding was also not observed as significantly different between the DOAC and VK in other studies that had approximately equal-sized groups [18–20]. Regarding the interruption of APT, most authors agree with the current literature, as it is considered not indicated. Additionally, because reversal of the irreversible effect of antiplatelet agents is not expected to occur until the life span of platelets has expired, discontinuation for dental surgery with increasing thromboembolic risk cannot be justified. Moreover, no significant differences in the frequency of postoperative bleeding after dental surgery can be observed when discontinuing or continuing APT [15, 21–23]. In this study, the patients did not interrupt the APT, and we did not observe significant postoperative bleeding events between the APT and DOAC/VK groups. However, the DOAC group within this study showed significant more postoperative bleeding events compared to APT or VK medication. The clinical advantages of DOACs consist of a rapid onset and known elimination half-life. Accordingly, if the patient's renal function is known, the dentist can assess the best time of interruption before dental surgery [24]. The recommendation derived from this information is to perform surgical interventions at a low active level, which means 12–24 h after the last intake of medication [25]. In a review, the authors concluded that interventions at low risk can be performed without interruption of the medication if unrestricted kidney function is present. Moreover, they recommend timing the surgery before the intake of medication and postponing it to 4 hours after the intervention if possible. In the case of a highrisk intervention, surgery should be performed at least 24h after the last intake of DOAC. High risk intervention is defined as multirooted teeth, whereas low-risk intervention is defined as the extraction of 1–3 teeth per quadrant or 2 implant placements [11]. In several studies, no significant postoperative bleeding was seen in DOAC groups compared to other AT groups, whereas the postoperative bleeding was significantly greater in DOAC groups than in healthy controls [11, 15]. This is not in accordance with our results within the DOAC group, where the 8 postoperative bleeding events occurred within 27 surgical interventions and were correlated to type A and C procedures. However, the intensity was of severity grade 1a–1c. The classification of severity is certainly very subjective and patient dependent, but it also allows postoperative bleeding to be realistically classified and objectified, since it is also the patient who suffers from more severe postoperative bleeding. In a study of 86 patients with DOAC and VK, the inserted bite swab was weighed to better objectify postoperative bleeding severity, and no postoperative bleeding was observed under these conditions [19]. However, saliva inflow itself also comes into play in this case. To exclude this bias, another study covered the salivary gland excretory ducts during the insertion of the bite swab. In this study, 84 patients with DOAC and VK were examined, and the weight of the bite swab was significantly higher in the VK patients [30]. Overall, these results are likely to be confounded by the proportion of vasoconstrictors in the procedure.

When reviewing the literature, it is noticeable that the definition of the type of surgical intervention and the severity of postoperative bleeding varies widely between different studies; thus, for general dentists, it remains difficult to assess a risk profile for patients under AT. This variation is particularly evident for cases in Germany, Austria and Switzerland, where questions about AT patients were answered as part of a study. The assessment of the bleeding risk of the different ATs (18% high risk for DOACs and 33% high risk for VCs) as well as the application of local haemostatic measures varies strongly [26]. No correlation between the type of intervention or number of extracted teeth and postoperative bleeding was observed in a prospective analysis with 838 patients that underwent DOAC, APT or VK therapy and the extraction of teeth or osteotomies [15]. This is consistent with a meta-analysis that did not show any correlation between the intervention type (extractions) and bleeding within ten included studies regarding APT in 535 patients [27]. Regarding other interventions, such as implant placements or lateral/vertical bone grafting in DOAC, APT or VK patients did not show a correlation between the invasiveness of the surgical procedure and postoperative bleeding frequencies [28]. According to a systematic review in VK patients, bleeding did not correlate with the extension of the surgical procedure [2]. Because of their particular design, these studies had in common that the surgeons evaluated the bleeding and assessed the patients at appropriate intervals, and this evaluation may be influenced by the fact that more experienced surgeons classify bleeding differently. In a retrospective study, however, the authors concluded that a significant correlation exists between the number of extracted teeth and postoperative bleeding in 1022 extracted teeth and vitamin-k anticoagulation patients [29]. Another retrospective study observed no significant correlation between the extraction of teeth or the osteotomy and postoperative bleeding in patients receiving vitamin-k anticoagulation or heparin bridging, but a significant impact of the number of teeth on postoperative bleeding events was seen in 520 tooth extractions in bridging patients [4]. This is in accordance with our results, as we observed more postoperative bleeding events in relation to type C procedures, whereas the distribution of interventions was quite balanced.

There are a few shortcomings of this study that need to be discussed. First, the subjective evaluation of the bleeding quality by the patient can lead to discrepancies between the groups in terms of the frequency and severity of postoperative bleeding. The amount of invasiveness of the surgical procedure can vary to a certain extent since the operations were performed by three different surgeons. Furthermore, the possible confounding factor of different effect levels of the DOACs with respect to renal function was not included in the evaluation.

In the current study, it can be concluded that postoperative bleeding events occur significantly more frequently in patients with anticoagulation therapy than in healthy patients, but the quality of bleeding shows significantly lower severity, even when a significant correlation with high-risk surgical interventions exists. It therefore appears reasonable to continue anticoagulation therapy perioperatively for small procedures and also the classified high-risk procedures within this study. Close interdisciplinary collaboration between oral surgeons and other medical specialists remains essential to minimise perioperative risks to patients, and the perioperative instruction of patients is crucial. Follow-up studies should include larger patient cohorts and investigate postoperative bleeding and its intensity under uniform risk classifications of surgical interventions.

## Data Availability

The datasets used and/or analyzed during the current study are available from the corresponding author on reasonable request.

## Abbreviations

AT: Anticoagulation therapy
VK: Vitamin K antagonist group
DOAC: Direct oral anticoagulants
APT: Antiplatelet therapy
CG: Control group
